# Insertion Success of the Laryngeal Tube in Emergency Airway Management

**DOI:** 10.1155/2016/3619159

**Published:** 2016-08-24

**Authors:** Michael Bernhard, André Gries, Alexandra Ramshorn-Zimmer, Volker Wenzel, Bjoern Hossfeld

**Affiliations:** ^1^Emergency Department, University Hospital of Leipzig, 04103 Leipzig, Germany; ^2^Department of Anaesthesiology and Critical Care Medicine, Innsbruck Medical University, 6020 Innsbruck, Austria; ^3^Department of Anaesthesiology and Intensive Care Medicine, Section Emergency Medicine, Federal Armed Forces Hospital, 89081 Ulm, Germany

## Abstract

*Background*. Emergency airway management (AM) is a priority when resuscitating critically ill or severely injured patients. The goal of this study was to determine the success rates of LT insertion during AM.* Methods*. Studies that included LT first-pass insertion (FPI) and overall-pass insertion (OPI) success by emergency medical services and in-hospital providers performing AM for emergency situations as well as for scheduled surgery published until July 2014 were searched systematically in Medline.* Results*. Data of 36 studies (*n* = 1,897) reported a LT FPI success by physicians of 82.5% with an OPI success of 93.6% (*p* < 0.001). A cumulative analysis of all 53 studies (*n* = 3,600) led to FPI and OPI success of 80.1% and 92.6% (*p* < 0.001), respectively. The results of 26 studies (*n* = 2,159) comparing the LT with the laryngeal mask airway (LMA) demonstrated a FPI success of 77.0 versus 78.7% (*p* = 0.36) and an OPI success of 92.2 versus 97.7% (*p* < 0.001).* Conclusion*. LT insertion failed in the first attempt in one out of five patients, with an overall failure rate in one out of 14 patients. When compared with the LT, the LMA had a cumulative 5.5% better OPI success rate.

## 1. Introduction

In critically ill or severely injured patients, efficacy of emergency airway management increased survival chances [1–3], independently of the setting in the emergency medical service, emergency departments, operating rooms, in-hospital resuscitation wards, or intensive care units [4, 5]. While tracheal intubation is considered to be the gold standard for emergency airway management [6–8], even experienced laryngoscopists may fail to intubate resulting in significant morbidity and mortality [9–11]. Since supraglottic airway devices were described to be effective and simple to use, with a steep learning curve among providers [12–14], they were introduced into emergency airway management guidelines in the prehospital and in-hospital setting as first-line device as well as a backup strategy after failed intubation attempts [2, 15–19].

In 1999, the laryngeal tube was introduced to the market by VBM. In the meantime, the design has been modified several times. Currently, five versions are available, standard LT reuseable (LT), standard LT single-use (LT-D), LT with suction lumen reuseable (LTS II), LT with suction lumen single-use (LTS-D), and intubating LT, to place an endotracheal tube secondary through the lumen (iTLS D).

Although the laryngeal tube is now about 15 years in service [20], there is little data about efficacy (e.g., high success rates) in emergency airway management in the field and outside of expert centers, where more experience with this device may yield better results [13]. This may be of importance since providers need to be aware of their own and procedure-related limitations while employing the laryngeal tube and need to recognize them in a dynamic situation when a patient is threatened by hypoxia and hypercapnea [21]. More knowledge about success rates such as first-pass and overall-pass insertion success rates with the laryngeal tube compared with other supraglottic devices such as the laryngeal mask may also improve patient safety. While individual skills and experience may differ, it is important to know which device may score best behind the gold standard tracheal intubation.

The goal of this study was to summarize the existing evidence of first-pass and overall-pass insertion success rates of laryngeal tube insertions in the out-of-hospital and in-hospital setting. Furthermore, we summarize the results of studies comparing the laryngeal tube with the laryngeal mask airway. Our hypothesis was that there would be no difference between devices being investigated.

## 2. Methods

### 2.1. Study Design

This study is a meta-analysis and review of the literature and does not involve the use of human subjects or medical records and thus does not require ethics approval.

### 2.2. Search Strategy

We searched Medline using the key words “laryngeal tube”, “laryngeal tube suction”, “first-pass success”, “first attempt”, and “overall success” as free-text terms and MeSH terms to identify relevant studies search strategy: [(“first insertion” AND “success”) OR (“insertion success”) OR (“attempt” OR “attempts” AND “success”) AND (“laryngeal tube” OR “laryngeal tube suction” OR “laryngeal tube suction-D” OR “LT” OR “LT-D” OR “LTS” OR (“LTSII”) OR “LTS-D”)]. We also hand-searched the references and bibliographies of the included and relevant articles and reviews. Citations were screened by all authors (*n* = 1,234), and those studies published upon 1999 meeting the predefined inclusion/exclusion criteria were then reviewed (*n* = 200). Relevant articles, as judged by all authors, were included for full review (*n* = 53). Disagreements were resolved through a consensus process with a third author. We did not search other databases such as Embase or Cochrane Central. As the rarely available studies were performed with the different modifications mentioned above (LT, LT-D, LTS II, and LTS-D), no observations of the single types were obtained, but all types were summarized as “LT.”

### 2.3. Inclusion and Exclusion Criteria

We considered studies in which emergency medical service personnel and in-hospital and prehospital physicians, regardless of speciality background and level of training, performed airway management in a given setting (e.g., emergency medical services, emergency department, intensive care unit, airway management during scheduled surgery, and in-hospital resuscitation in wards) using the laryngeal tube. We excluded paediatric patients. Articles published in English and German were included. All case reports, case reviews, systematic reviews, letters to the editor, and animal as well as cadaver or mannequin studies were excluded.

### 2.4. Outcome Measures

The primary outcome measures were the first-pass and overall-pass insertion success rates of laryngeal tube insertion. Secondary outcome measures were first-pass and overall-pass insertion success rates of laryngeal mask airway (as another often used supraglottic airway device) in studies comparing laryngeal tube and laryngeal mask airway. The criterion for “successful insertion” in the included studies was adequacy of ventilation observing the presence of end-tidal carbon dioxide waveforms, chest movement, and oxygen saturation.

### 2.5. Statistical Analysis

The statistical analysis was performed with the statistical software package R (version 2.15.1). The results of first-pass and overall-pass insertion success rates of the included studies were summarized and reported in detail. Manual calculations of unadjusted effects estimates (odds ratio, OR) were performed, and the odd ratios were pooled. 95% confidence intervals (CI) for each study and over all studies were calculated. If the success rate was 100%, it was assumed that the lower confidence interval (CI) was reached and that the next attempt would fail. In those cases, the CI was marked in the results tables. We used a fixed effects model. Group comparisons of frequencies were performed using the Chi^2^ test. For all randomized controlled trials, the odds ratio was calculated and presented with confidence intervals in a forest plot.

## 3. Results

**Figure 1:**
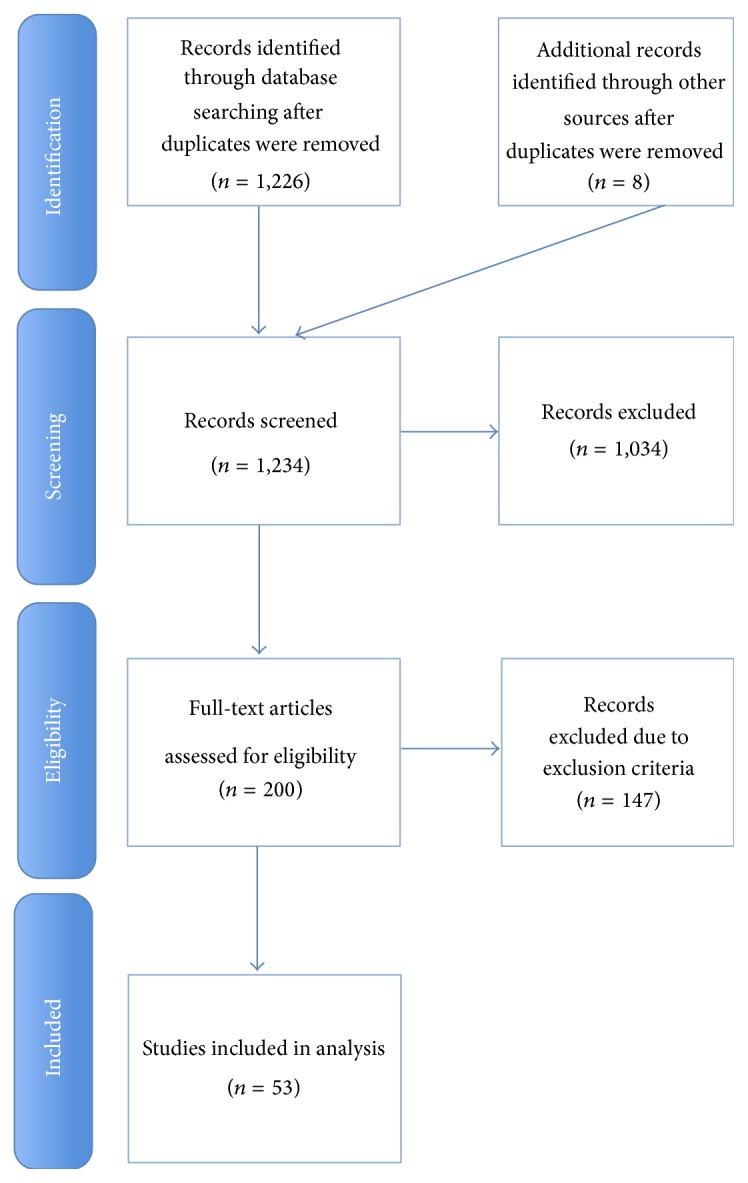
Flow diagram for selection of trials.

This search identified 1,234 citations, of which 1034 were excluded (Figure 1). We reviewed the full text of 200 studies, of which 53 studies met inclusion criteria.

### 3.1. First-Pass and Overall Success of Laryngeal Tube Insertion

Physicians employed the laryngeal tube during scheduled anaesthesia in 36 studies [22–56] with a total of 1,897 patients: the first-pass insertion success rate of these providers was 82.5% (1,452/1,760, 95% CI: 80.7%–84.3%), and the overall-pass insertion success rate was 93.6% (1,594/1,703) (95% CI: 92.4%–94.8%, *p* < 0.001) (Table 1). Paramedics, paramedic students, and BLS nurses employed the laryngeal tube in an in-hospital setting in 4 studies [57–60] including 151 patients. In this group, the first-pass insertion success rate was 53.2% (25/47, 95% CI: 38.9%–67.5%) and the overall-pass insertion success rate was 87.4% (132/151) (95% CI: 82.1%–92.7%, *p* < 0.001) (Table 2). Physicians, paramedics, first responders, and nurses employed the laryngeal tube outside the hospital in 13 studies [61–73] with 1,552 patients reporting the first-pass and/or overall-pass insertion success rates of laryngeal tube insertion to be 78.1% (1,212/1,552, 95% CI: 76.0%–80.2%) and the overall-pass insertion success rate was 91.8% (1,272/1,385) (95% CI: 90.4%–93.3%, *p* < 0.001) (Table 3). In the cumulative analysis with all 53 studies [22–73] including 3,600 patients, the first-pass insertion success rate of the laryngeal tube was 80.1% (2,689/3,359) and the overall-pass insertion success rate was 92.6% (2,938/3,239) (*p* < 0.001).

### 3.2. Laryngeal Tube versus Laryngeal Mask Airway

**Figure 2:**
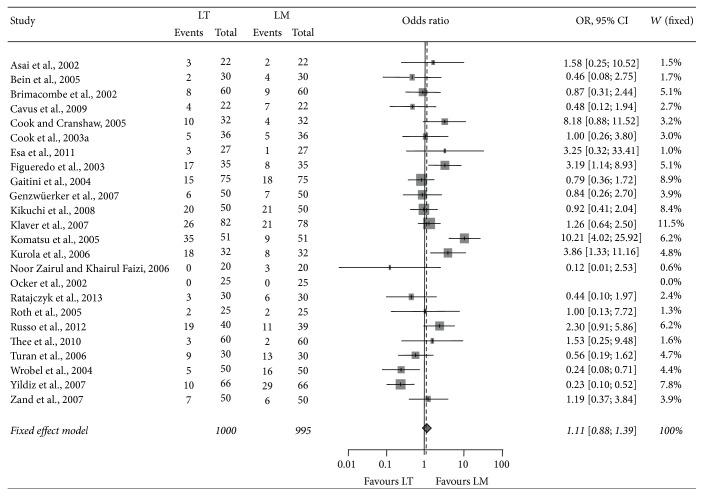
Forest plot of first-pass insertion failure (LT laryngeal tube, LM laryngeal mask).

**Figure 3:**
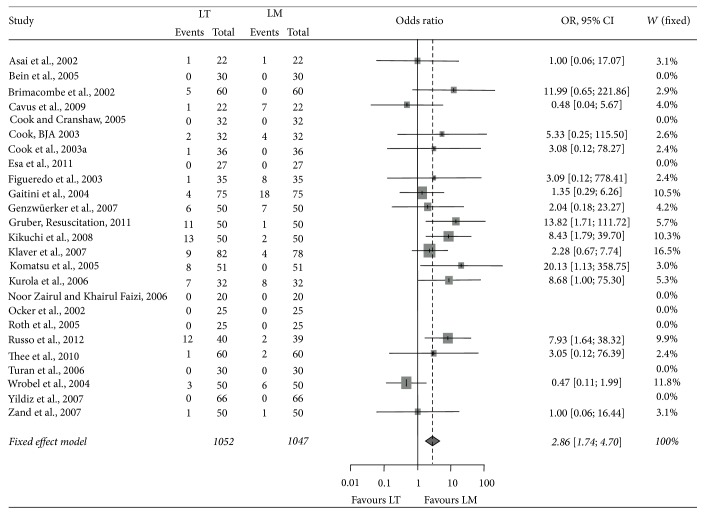
Forest plot of overall-insertion failure (LT laryngeal tube, LM laryngeal mask).

In 26 studies including 2,159 patients comparing the laryngeal tube with the laryngeal mask airway in the operating room (Table 4), the first-pass insertion success rates for the laryngeal tube versus the laryngeal mask airway were 77.0% (770/1,000) versus 78.7% (783/995) (*p* = 0.36), and the overall-pass insertion success rate was 92.2% (970/1,052) versus 97.7 (1,023/1,047) (*p* < 0.001) (Table 4). The pooled OR across all studies was 1.11 (95% CI: 0.88–1.39) for the first-pass insertion failure, indicating comparable success rates of both devices (Figure 2). The pooled OR across all studies was 2.86 (95% CI: 1.74–4.70) for overall-pass insertion failure, indicating lower failure rate for laryngeal mask airway when compared to laryngeal tube (Figure 3).

## 4. Discussion

This study included 53 studies from 17 different countries with a pooled sample size of 3,600 patients, which is the largest summarized data set assessing laryngeal tube insertion success rates at the current time. Overall-pass insertion success rate employing the laryngeal tube was 92.6%, with the laryngeal mask insertion being significantly better at 97.7%.

Our results were contrary to some findings in the literature from smaller meta-analysis, which found an overall success rate of the laryngeal tube insertions (*n* = 150) of 96.5% and of the laryngeal mask airway (*n* = 3,829) of 87.4% [74]. The different overall-pass insertion success rates of laryngeal tube insertion in comparison to laryngeal mask insertion may have the following reasons: first, anaesthetists are well trained in laryngeal mask airway usage in the operating room providing anaesthesia and airway management on a daily basis [14]; emergency airway management is then just an extrapolation of daily routine with this device. For the laryngeal tube, this remains a dilemma since this supraglottic airway device is usually not being routinely employed in the operating room at all [75], which subsequently may explain the low laryngeal tube first-pass insertion success rate (77.7%) and overall-pass insertion success rate (92.2%) in the prehospital setting. Second, if a device is not being employed routinely in the operating room, anticipated success rates in an emergency airway management situation simply have to be worse compared to the daily routine device as a difficult situation has to be managed with an unacquainted device. Third, significant problems of laryngeal tube insertion have been reported, including incorrect positioning in prehospital and in-hospital settings, which may contribute to the phenomenon of inadvertent complications [21, 76]. A prospective study reported a complication rate of 52% in 189 patients being managed with the laryngeal tube in the prehospital setting [76, 77]: laryngeal tube-associated problems related to excessive cuff pressure, tongue swelling (39%), hypoxic cardiac arrest (2%), massive stomach distension with ventilation difficulty (11%), and bleeding from soft tissue of the upper airway (2%) [76, 77].

A range of the previously mentioned complications were reported in the underlying studies. However, the reporting style of adverse events in those studies was unstructured and maybe incomplete. Therefore, those results have to be interpreted with caution, and a robust conclusion may not be drawn from those elective in-hospital studies.

Taken together, the laryngeal tube may not be as simple as anticipated, and lack of its daily usage in airway management including emergency cases may hamper success especially when hypoxic and hypercarbic patients need it the most [21].

The successful insertion of a supraglottic airway is important; however, this may not reflect successful ventilation with the given device during emergency conditions in all cases. Carlson et al. [78] described in a human unembalmed cadaver model that the required axial dislodgment force for a combitube required in this study is twice as much as for the endotracheal tube; and the laryngeal tube and the laryngeal mask airway dislodgment forces were similar to those of the endotracheal tube. Thereby, using second-generation supraglottic airway devices may optimize the seal and reduce the possibility of accidental dislodgement [76]. The current guidelines of the European Resuscitation Council [2] report rates of successful ventilation during cardiopulmonary resuscitation of 71–90% for the laryngeal mask airway in comparison to the combitube with 79–98% and no reported percentages for the laryngeal tube. However, cardiac arrest is only one of the prehospital emergencies requiring emergency airway management; other acute settings (e.g., trauma, respiratory insufficiency) need emergency airway management as well. The included studies in this review were not able to answer the question of which supraglottic airway is superior to another in means of successful ventilation in emergency circumstances.

A meta-analysis and systematic review [79] assessing the survival rates of patients suffering from out-of-hospital cardiac arrest being managed with tracheal intubation versus supraglottic airway devices versus basic airway management suggested decreases in survival rates for out-of-hospital cardiac arrest victims treated by the EMS with advanced airway interventions. Unfortunately, this and other similar studies may fall prey to their design; namely, advanced airway management may simply trail ongoing resuscitation efforts. As such, a patient regaining spontaneous circulation quickly may simply not need tracheal intubation, while a patient undergoing prolonged resuscitation efforts with a slim survival chance is earmarked with advanced airway management. In this case, advanced airway management would simply be a surrogate parameter for bad outcome, but not the problem per se.

## 5. Limitations

This study has several limitations. First, the review pools existing evidence and is thus dependent on quality of reported data. Reviews of randomized controlled trials provide the strongest evidence, which only was the case in the studies comparing the laryngeal tube with the laryngeal mask airway, but not in studies investigating the laryngeal tube alone. Moreover, typical for advanced airway studies, the success rates are very high, and for the distributions these proportions may be much skewed towards the high end. Therefore, as the success proportions approach hundred percent, the variance of the study is squeezed towards zero, and, therefore, the study with the high success proportion may receive a disproportionately large weight when the inverse variance method such as fixed effects model is used. So, the pooled odds ratios of this might overestimate the effect [80]. Further, we excluded small case series and letters to the editor, which is a strength of our analysis; also, we excluded mannequin studies as this setting cannot be extrapolated directly to emergency airway management [81]. Also, the majority of data was extracted from studies with anaesthetists who are experienced in maintaining the airway, thus featuring a possible positive bias for success rates of the chosen airway device. In contrast, results from mixed providers including physicians and mostly paramedics performing laryngeal tube insertion revealed lower laryngeal tube insertion success rates, which further decreased when paramedics during training only inserted laryngeal tubes in the operating room, thus illustrating a learning curve. The different experience of the practitioners must be discussed; namely, some providers may have been much more skilled in using the laryngeal mask airway than the laryngeal tube and several of the comparative studies are thus a comparison of skill levels and not of devices per se. Dividing the comparative studies of the laryngeal tube and the laryngeal mask airway according to the performer level demonstrated that anaesthetists have a higher first-pass and overall-pass insertion success rate for both devices in comparison to nonanaesthetists and that first-pass and overall-pass insertion success rates of nonanaesthetists for the laryngeal tube are inferior to the laryngeal mask airway (Table 2). These results suggest that there is a device and also a provider dependent influence on success rates. This analysis, however, is limited by the small number of patients in the studies of laryngeal tube insertion by nonanaesthetists (*n* = 82). Similar to a historic discussion about the combitube, airway device-related complications may be significantly more related to the training with airway devices than to the airway devices themselves [82]. Accordingly, it is possible that the full potential of the laryngeal tube has not been detected yet as teaching with it may remain insufficient and infrequent.

## 6. Conclusions

Laryngeal tube (including its newer modifications) insertion failed in the first attempt in one out of five patients, with an overall failure rate in one out of 14 patients. When compared with the laryngeal tube, the laryngeal mask airway had a cumulative 5.5% better overall-pass insertion success rate.

## Figures and Tables

**Table 1:** Success rates of laryngeal tubes in in-hospital airway management by physicians.

Authors	Study	Device	Healthcare provider	*n*	FPIS		OPIS	
					[*n*, (%)]	95% CI	[*n*, (%)]	95% CI
Ratajczyk et al., 2013 [48]	p, in-hospital, elective	LT	Physicians	30	27, 90.0	79.3%–100.0%	NS	—
Ozgul et al., 2013 [47]	p, in-hospital, elective	LTS II	Physicians	80	75, 93.8	88.4%–99.1%	80, 100.0	96.4%–100.0%
Russo et al., 2012 [50]	p, in-hospital, elective	LTS-D	Physicians	40	21, 52.5	37.0%–68.0%	28, 70.0	55.8%–84.2%
Esa et al., 2011 [35]	p, in-hospital, elective	LTS II	Physicians	27	24, 88.9	77.0%–100.0%	27, 100.0	89.6%–100.0%
Schalk et al., 2011 [51]	p, in-hospital, elective	LTS-D	Physicians	105	NS	—	84, 80.0	72.3%–87.7%
Amini et al., 2010 [23]	p, in-hospital, elective	LTS-D/LTS II	Physicians	60	54, 90.0	82.4%–97.6%	58, 96.0	92.1%–100.0%
Thee et al., 2010 [52]	p, in-hospital, elective	LTS-D/LTS II	Physicians	60	57, 95.0	89.5%–100.0%	59, 98.3	95.1%–100.0%
Klaver et al., 2007 [42]	p, in-hospital, elective	LTS	Physicians	82	56, 68.0	58.2%–78.4%	NS	—
Cavus et al., 2009 [29]	p, in-hospital, elective	LTS	Physicians	22	18, 82.0	65.7%–97.9%	21, 96.0	86.8%–100.0%
Kikuchi et al., 2008 [41]	p, in-hospital, elective	LTS II	Physicians	50	30, 60.0	46.4%–73.6%	37, 74.0	61.8%–86.2%
Zand et al., 2007 [56]	p, in-hospital, elective	LTS	Physicians	50	43, 86.0	76.4%–95.6%	49, 98.0	94.1%–100.0%
Yildiz et al., 2007 [55]	p, in-hospital, elective	LT	Physicians	66	56, 84.8	76.2%–93.5%	66, 100.0	95.6%–100.0%
Genzwuerker et al., 2007 [39]	p, in-hospital, elective	LTS II	Physicians	50	44, 88.0	79.0%–97.0%	48, 96.0	90.6%–100.0%
Amini et al., 2007 [22]	p, in-hospital, elective	LT-D, LT-R	Physicians	100	93, 93.0	88.0%–98.0%	98, 98.0	95.3%–100.0%
Mihai et al., 2007 [44]	p, in-hospital, elective	LTS II	Physicians	100	71, 71.0	62.1%–79.9%	100, 100.0	97.1%–100.0%
Noor Zairul and Khairul Faizi, 2006 [45]	p, in-hospital, elective	LT	Physicians	20	20, 100.0	86.1%–100.0%	20, 100.0	86.1%–100.0%
Turan et al., 2006 [53]	p, in-hospital, elective	LT	Physicians	30	21, 69.0	53.6%–86.4%	23, 76.0	61.5%–91.8%
Bein et al., 2005 [27]	p, in-hospital, elective	LTS	Physicians	30	28, 93.3	84.4%–100.0%	30, 100.0	90.6%–100.0%
Cook and Cranshaw, 2005 [30]	p, in-hospital, elective	LTS	Physicians	32	22, 68.8	52.7%–84.8%	NS	—
Roth et al., 2005 [49]	p, in-hospital, elective	LTS	Physicians	25	23, 92.0	81.4%–100.0%	25, 100.0	88.8%–100.0%
Komatsu et al., 2005 [43]	p, in-hospital, elective	LT	Physicians	51	16, 31.4	18.6%–44.1%	42, 82.4	71.9%–92.8%
Gaitini et al., 2004 [38]	p, in-hospital, elective	LTS	Physicians	75	60, 80.0	70.9%–89.1%	71, 94.7	89.6%–99.8%
Wrobel et al., 2004 [54]	p, in-hospital, elective	LT	Physicians	50	45, 90.0	81.7%–98.3%	47, 94.0	87.4%–100.0%
Figueredo et al., 2003 [36]	p, in-hospital, elective	LT	Physicians	35	18, 51.0	34.9%–68.0%	24, 97.0	53.2%–84.0%
Cook et al., 2003 [31]	p, in-hospital, elective	LT	Physicians	32	NS	—	30, 93.8	85.4%–100.0%
Cook et al., 2003 [32]	p, in-hospital, elective	LT	Physicians	36	31, 86.1	74.8%–97.4%	35, 97.2	91.9%–100.0%
Genzwürker et al., 2003 [40]	p, in-hospital, elective	LTS	Physicians	30	27, 90.0	79.3%–100.0%	29, 96.7	90.2%–100.0%
Gaitini et al., 2003 [37]	p, in-hospital, elective	LT	Physicians	175	159, 94.0	86.6%–95.1%	169, 96.6	93.9%–99.3%
Asai et al., 2003 [26]	p, in-hospital, elective	LT	Physicians	100	90, 90.0	84.1%–95.9%	97, 97.0	93.7%–100.0%
Dörges et al., 2003 [33]	p, in-hospital, elective	LT	Physicians	32	32, 100.0	91.1%–100.0%	32, 100.0	91.1%–100.0%
Figueredo et al., 2003 [36]	p, in-hospital, elective	LT	Physicians	35	18, 51.0	34.9%–68.0%	34, 97.0	91.6%–100.0%
Ocker et al., 2002 [46]	p, in-hospital, elective	LT	Physicians	25	25, 100.0	88.8%–100.0%	25, 100.0	88.8%–100.0%
Brimacombe et al., 2002 [28]	p, in-hospital, elective	LT	Physicians	60	52, 87.0	78.1%–95.3%	55, 92.0	84.7%–98.7%
Asai et al., 2002 [24]	p, in-hospital, elective	LT	Physicians	22	19, 86.4	72.0%–100.0%	21, 95.5	86.8%–100.0%
Asai et al., 2000 [25]	p, in-hospital, elective	LT	Physicians	50	47, 94.0	87.4%–100.0%	NS	—
Dörges et al., 2000 [34]	p, in-hospital, elective	LT	Physicians	30	30, 100.0	90.6%–100.0%	30, 100.0	90.6%–100.0%

			*Cumulative sum (n*)	*1897*	*1452/1760*		*1594/1703*	
			*(%)*		*82.5*	*80.7%–84.3%*	*93.6*	*92.4%–94.8%*

NS: not stated; p: prospective; r: retrospective; elective: nonemergency patient; FPIS: first-pass insertion success; OPIS: overall-pass insertion success.

If the success rate was 100%, it was assumed that the lower confidence interval (CI) was reached and that the next attempt would fail.

**Table 2:** Success rates of laryngeal tubes in in-hospital airway management by different providers (physicians, paramedics, basic life support nurses, and paramedic students).

Authors	Study	Device	Healthcare provider	*n*	FPIS		OPIS	
					[*n*, (%)]	95% CI	[*n*, (%)]	95% CI
Schalk et al., 2008 [60]	p, in-hospital, elective	LTS-D	Physicians, paramedics	54	NS	—	53, 98.1	94.6%–100.0%
Gruber et al., 2014 [57]	p, in-hospital, elective	LTS-D	BLS nurses	50	NS	—	39, 78.0	66.5%–89.5%
Kurola et al., 2005 [59]	p, in-hospital, elective	LT	Paramedics	15	11, 73.3	51.0%–95.7%	15, 100.0	81.9%–100.0%
Kurola et al., 2006 [58]	p, in-hospital, elective	LT	Paramedics students	32	14, 43.8	26.6%–60.9%	25, 78.1	63.8%–92.4%

			*Cumulative sum (n*)	*151*	*25, 47*		*132, 151*	
			*(%)*		*53.2*	*38.9%–67.5%*	*87.4*	*82.1%–92.7%*

NS: not stated; p: prospective; r: retrospective; elective: nonemergency patient; BLS: basic life support; FPIS: first-pass insertion success; OPIS: overall-pass insertion success. If the success rate was 100%, it was assumed that the lower confidence interval (CI) was reached and that the next attempt would fail.

**Table 3:** Success rates of laryngeal tubes in prehospital airway management by different providers (first responder, physicians, paramedics, registered nurses, and physicians).

Authors	Study	Device	Healthcare provider	*n*	FPIS		OPIS	
					[*n*, (%)]	95% CI	[*n*, (%)]	95% CI
Länkimäki et al., 2013 [66]	p, prehospital, OOHCA	LT	First responder	64	46, 71.9	60.9%–82.9%	59, 92.2	85.6%–98.8%
Frascone et al., 2013 [61]	p, prehospital, emerg.	LTS-D	Paramedics/RN	38	29, 76.3	62.8%–89.8%	32, 84.2	72.6%–95.8%
Müller et al., 2013 [67]	p, prehospital, OOHCA	LT-D	Paramedics	130	108, 83.1	76.6%–89.5%	121, 93.1	88.7%–97.4%
Schalk et al., 2012 [69]	p, prehospital, emerg.	LTS-D	Physicians/paramedics	303	223, 73.6	68.6%–78.6%	296, 97.8	96.0%–99.4%
Sunde et al., 2012 [72]	r, prehospital, OOHCA	LT, NS	Paramedics	347	258, 74.4	69.8%–78.9%	296, 85.3	81.6%–89.0%
Gahan et al., 2011 [63]	r, prehospital, OOHCA	LT-D	First responder	167	147, 88.0	83.1%–92.9%	NS	—
Frascone et al., 2011 [62]	p, prehospital, emerg.	LTS-D	Paramedics	128	86, 67.2	59.1%–75.3%	103, 80.5	73.6%–87.3%
Schalk et al., 2011 [71]	p, prehospital, trauma	LTS-D	Physicians/paramedics	57	50, 87.7	79.2%–96.2%	56, 98.2	94.8%–100.0%
Schalk et al., 2010 [70]	p, prehospital, emerg.	LT-D/LTS-D	Physicians/paramedics	157	123, 78.3	71.9%–84.8%	152, 96.8	94.1%–99.6%
Wiese et al., 2009 [73]	p, prehospital, emerg.	LT-D	Paramedics	92	85, 92.4	87.0%–97.8%	92, 100.0	96.8%–100.0%
Russi et al., 2008 [68]	p, prehospital, emerg.	King LT	Paramedics	13	12, 92.3	77.8%–100.0%	13, 100.0	79.4%–100.0%
Guyette et al., 2007 [64]	r, prehospital, emerg.	LT-D	Paramedics/nurses	26	24, 92.3	82.1%–100.0%	26, 100.0	89.2%–100.0%
Kette et al., 2005 [65]	p, prehospital, OOHCA	LT	Nurses	30	21, 70.0	53.6%–86.4%	26, 86.7	74.5%–98.8%

			*Cumulative sum (n*)	*1552*	*1212/1552*		*1272/1385*	
			*(%)*		*78.1*	*76.0%–80.2%*	*91.8*	*90.4%–93.3%*

NS: not stated; p: prospective; r: retrospective; OOHCA: out-of-hospital cardiac arrest; emerg.: emergency/no cardiac arrest; FPIS: first-pass insertion success; OPIS: overall-pass insertion success; RN: registered nurses. If the success rate was 100%, it was assumed that the lower confidence interval (CI) was reached and that the next attempt would fail.

**Table 4:** Comparison of insertion success rates of laryngeal tubes with laryngeal mask airways.

Authors	Study design	Healthcare provider	Patients	Type of laryngeal tube	FPIS (*n*)	OPIS (*n*)	Type of laryngeal mask airway	FPIS (*n*)	OPIS (*n*)
Asai et al., 2002 [24]	p, randomized	Anaesthetists	Adult, ASA I/II	LT	19/22	21/22	cLMA	20/22	21/22
Bein et al., 2005 [27]	p, randomized	Anaesthetists	Adult, ASA I/II/III	LTS	28/30	30/30	PLMA	26/30	30/30
Brimacombe et al., 2002 [28]	p, randomized	Anaesthetists	Adult, ASA I/II	LT	52/60	55/60	PLMA	51/60	60/60
Cavus et al., 2009 [29]	p, randomized	Anaesthetists	Adult, ASA I/II	LTS II	18/22	21/22	PLMA	15/22	20/22
Cook and Cranshaw, 2005 [30]	p, randomized	Anaesthetists	Adult, ASA I/III	LTS	22/32	32/32	PLMA	28/32	32/32
Cook et al., 2003 [31]	p, randomized	Anaesthetists	Adult, ASA I–III	LT	NS	30/32	PLMA	NS	32/32
Cook et al., 2003 [32]	p, randomized	Anaesthetists	Adult, ASA I/II	LT	31/36	35/36	cLMA	31/36	36/36
Esa et al., 2011 [35]	p, randomized	Anaesthetists	Adult, ASA I/II	LTS II	24/27	27/27	PLMA	26/27	27/27
Figueredo et al., 2003 [36]	p, randomized	Anaesthetists	Adult, ASA I/II	LTS	18/35	34/35	PLMA	27/35	35/35
Gaitini et al., 2004 [38]	p, randomized	Anaesthetists	Adult, ASA I/II	LTS	60/75	71/75	PLMA	57/75	72/75
Genzwuerker et al., 2007 [39]	p, randomized	Anaesthetists	Adult, ASA I/II/III	LTS II	44/50	48/50	PLMA	43/50	49/50
Gruber et al. [57], Resuscitation, 2011	p, randomized	BLS nurses	Adult, ASA I/II	LTS-D	NS	39/50	LMAS	NS	49/50
Kikuchi et al., 2008 [41]	p, randomized	Anaesthetists	Adult, ASA I/II	LTS II	30/50	37/50	PLMA	29/50	48/50
Klaver et al., 2007 [42]	p, randomized	Anaesthetists	Adult, ASA I/II	LTS	56/82	73/82	PLMA	57/78	74/78
Komatsu et al., 2005 [43]	p, randomized	Anaesthetists	Adult, ASA I/II	LT	16/51	43/51	ILMA	42/51	51/51
Kurola et al., 2006 [58]	p, random order	Para. students	Adult, ASA I/II	LT	14/32	25/32	ILMA	24/32	31/32
Noor Zairul and Khairul Faizi, 2006 [45]	p, randomized	Anaesthetists	Adult, ASA I/II	LT	20/20	20/20	cLMA	17/20	20/20
Ocker et al., 2002 [46]	p, randomized	Anaesthetists	Adult, ASA I/II	LT	25/25	25/25	cLMA	25/25	25/25
Ratajczyk et al., 2013 [48]	p, randomized	Anaesthetists	Adult, ASA I/II	LT	27/30	NS	cLMA	24/30	NS
Roth et al., 2005 [49]	p, randomized	Anaesthetists	Adult, ASA I/II	LTS	23/25	25/25	PLMA	23/25	25/25
Russo et al., 2012 [50]	p, randomized	Anaesthetists	Adult, ASA I/II	LTS-D	21/40	28/40	LMAS	28/39	37/39
Thee et al., 2010 [52]	p, randomized	Anaesthetists	Adult, ASA I/II/III	LTS II/D	57/60	59/60	ILMA/D	58/60	60/60
Turan et al., 2006 [53]	p, randomized	Anaesthetists	Adult, ASA I/II	LT	21/30	30/30	cLMA	17/30	30/30
Wrobel et al., 2004 [54]	p, randomized	Anaesthetists	Adult, ASA I/II/III	LT	45/50	47/50	cLMA	34/50	44/50
Yildiz et al., 2007 [55]	p, randomized	Anaesthetists	Adult, ASA I/II	LT	56/66	66/66	cLMA	37/66	66/66
Zand et al., 2007 [56]	p, randomized	Anaesthetists	Adult, ASA I/II	LTS	43/50	49/50	PLMA	44/50	49/50

			*Cumulative sum* *(%)*		*770/1000* *77.0*	*970/1052* *92.2*		*783/995* *78.7*	*1023/1047* *97.7*

NS: not stated; p: prospective; FPIS: first-pass insertion success; BLS: basic life support; para: paramedic; ASA: American Society of Anesthesiologists classification; OPIS: overall-pass insertion success; LT: laryngeal tube; LTS: laryngeal tube suction; cLMA: classic laryngeal mask; PLMA: ProSeal laryngeal mask; LMAS: laryngeal mask supreme; ILMA: intubating laryngeal mask; D: disposable.

## References

[1] Berlac P., Hyldmo P. K., Kongstad P. (2008). Pre-hospital airway management: guidelines from a task force from the Scandinavian Society for Anaesthesiology and Intensive Care Medicine. *Acta Anaesthesiologica Scandinavica*.

[2] Deakin C. D., Nolan J. P., Soar J. (2010). European Resuscitation Council Guidelines for Resuscitation 2010 Section 4. Adult advanced life support. *Resuscitation*.

[3] Paal P., Herff H., Mitterlechner T. (2010). Anaesthesia in prehospital emergencies and in the emergency room. *Resuscitation*.

[4] Cook T. M., Woodall N., Frerk C., Fourth National Audit Project (2011). Major complications of airway management in the UK: results of the Fourth National Audit Project of the Royal College of Anaesthetists and the Difficult Airway Society. Part 1: anaesthesia. *British Journal of Anaesthesia*.

[5] Cook T. M., Woodall N., Harper J., Benger J. (2011). Major complications of airway management in the UK: results of the Fourth National Audit Project of the Royal College of Anaesthetists and the Difficult Airway Society—part 2: intensive care and emergency departments. *British Journal of Anaesthesia*.

[6] Komatsu R., Kasuya Y., Yogo H. (2010). Learning curves for bag-and-mask ventilation and orotracheal intubation: an application of the cumulative sum method. *Anesthesiology*.

[7] Konrad C., Schüpfer G., Wietlisbach M., Gerber H. (1998). Learning manual skills in anesthesiology: is there a recommended number of cases for anesthetic procedures?. *Anesthesia and Analgesia*.

[8] Bernhard M., Mohr S., Weigand M. A., Martin E., Walther A. (2012). Developing the skill of endotracheal intubation: implication for emergency medicine. *Acta Anaesthesiologica Scandinavica*.

[9] Timmermann A., Russo S. G., Eich C. (2007). The out-of-hospital esophageal and endobronchial intubations performed by emergency physicians. *Anesthesia and Analgesia*.

[10] Von Goedecke A., Herff H., Paal P., Dörges V., Wenzel V. (2007). Field airway management disasters. *Anesthesia & Analgesia*.

[11] Gries A., Zink W., Bernhard M., Messelken M., Schlechtriemen T. (2006). Realistic assessment of the physican-staffed emergency services in Germany. *Anaesthesist*.

[12] Timmermann A. (2011). Supraglottic airways in difficult airway management: successes, failures, use and misuse. *Anaesthesia*.

[13] Ostermayer D. G., Gausche-Hill M. (2014). Supraglottic airways: the history and current state of prehospital airway adjuncts. *Prehospital Emergency Care*.

[14] Mohr S., Weigand M. A., Hofer S. (2013). Developing the skill of laryngeal mask insertion: prospective single center study. *Anaesthesist*.

[15] Apfelbaum J. L., Hagberg C. A., Caplan R. A. (2013). Practice guidelines for management of the difficult airway: an updated report by the American Society of Anesthesiologists Task Force on Management of the Difficult Airway. *Anesthesiology*.

[16] Heidegger T., Gerig H. J., Henderson J. J. (2005). Strategies and algorithms for management of the difficult airway. *Best Practice and Research: Clinical Anaesthesiology*.

[17] Henderson J. J., Popat M. T., Lotto I. P., Pearce A. C. (2004). Difficult airway society guidelines for management of the unanticipated difficult intubation. *Anaesthesia*.

[18] Herff H., Wenzel V., Lockey D. (2009). Prehospital intubation: the right tools in the right hands at the right time. *Anesthesia and Analgesia*.

[19] Neumar R. W., Otto C. W., Link M. S. (2010). Part 8: adult advanced cardiovascular life support: 2010 American Heart Association Guidelines for Cardiopulmonary Resuscitation and Emergency Cardiovascular Care. *Circulation*.

[20] Agro F., Cataldo R., Alfano A., Galli B. (1999). A new prototype for airway management in an emergency: the laryngeal tube. *Resuscitation*.

[21] Bernhard M., Beres W., Timmermann A. (2014). Prehospital airway management using the laryngeal tube. An emergency department point of view. *Anaesthesist*.

[22] Amini A., Zand F., Sadeghi S. E. (2007). A comparison of the disposable vs the reusable laryngeal tube in paralysed adult patients. *Anaesthesia*.

[23] Amini A., Zand F., Maghbooli M. (2010). Disposable versus reusable laryngeal tube suction for ventilation in patients undergoing laparoscopic cholecystectomy. *Revista Brasileira de Anestesiologia*.

[24] Asai T., Kawashima A., Hidaka I., Kawachi S. (2002). The laryngeal tube compared with the laryngeal mask: insertion, gas leak pressure and gastric insufflation. *British Journal of Anaesthesia*.

[25] Asai T., Murao K., Shingu K. (2000). Efficacy of the laryngeal tube during intermittent positive-pressure ventilation. *Anaesthesia*.

[26] Asai T., Shingu K., Cook T. (2003). Use of the laryngeal tube in 100 patients. *Acta Anaesthesiologica Scandinavica*.

[27] Bein B., Carstensen S., Gleim M. (2005). A comparison of the proseal laryngeal mask airway, the laryngeal tube S and the oesophageal-tracheal combitube during routine surgical procedures. *European Journal of Anaesthesiology (EJA)*.

[28] Brimacombe J., Keller C., Brimacombe L. (2002). A comparison of the laryngeal mask airway ProSeal^*™*^ and the laryngeal tube airway in paralyzed anesthetized adult patients undergoing pressure-controlled ventilation. *Anesthesia and Analgesia*.

[29] Cavus E., Deitmer W., Francksen H. (2009). Laryngeal tube S II, ProSeal laryngeal mask, and EasyTube during elective surgery: a randomized controlled comparison with the endotracheal tube in nontrained professionals. *European Journal of Anaesthesiology*.

[30] Cook T. M., Cranshaw J. (2005). Randomized crossover comparison of ProSeal® Laryngeal Mask Airway with Laryngeal Tube Sonda® during anaesthesia with controlled ventilation. *British Journal of Anaesthesia*.

[31] Cook T. M., McKinstry C., Hardy R., Twigg S. (2003). Randomized crossover comparison of the ProSeal*™* laryngeal mask airway with the Laryngeal Tube® during anaesthesia with controlled ventilation. *British Journal of Anaesthesia*.

[32] Cook T. M., McCormick B., Asai T. (2003). Randomized comparison of laryngeal tube with classic laryngeal mask airway for anaesthesia with controlled ventilation. *British Journal of Anaesthesia*.

[33] Dörges V., Ocker H., Wenzel V., Steinfath M., Gerlach K. (2003). The laryngeal tube S: a modified simple airway device. *Anesthesia and Analgesia*.

[34] Dörges V., Ocker H., Wenzel V., Schmucker P. (2000). The laryngeal tube: a new simple airway device. *Anesthesia and Analgesia*.

[35] Esa K., Azarinah I., Muhammad M., Helmi M. A., Jaafar M. Z. (2011). A comparison between laryngeal tube suction II airway and Proseal laryngeal mask airway in laparoscopic sugery. *Medical Journal of Malaysia*.

[36] Figueredo E., Martínez M., Pintanel T. (2003). A comparison of the ProSeal*™* laryngeal mask and the Laryngeal Tube® in spontaneously breathing anesthetized patients. *Anesthesia and Analgesia*.

[37] Gaitini L. A., Vaida S. J., Somri M. (2003). An evaluation of the laryngeal tube during general anesthesia using mechanical ventilation. *Anesthesia & Analgesia*.

[38] Gaitini L. A., Vaida S. J., Somri M., Yanovski B., Ben-David B., Hagberg C. A. (2004). A randomized controlled trial comparing the Proseal laryngeal mask airway with the laryngeal tube suction in mechanically ventilated patients. *Anesthesiology*.

[39] Genzwuerker H. V., Altmayer S., Hinkelbein J., Gernoth C., Viergutz T., Ocker H. (2007). Prospective randomized comparison of the new laryngeal tube suction LTS II and the LMA-ProSeal for elective surgical interventions. *Acta Anaesthesiologica Scandinavica*.

[40] Genzwürker H., Finteis T., Hinkelbein J., Ellinger K. (2003). First clinical experiences with the new LTS. A laryngeal tube with an oesophageal drain. *Anaesthesist*.

[41] Kikuchi T., Kamiya Y., Ohtsuka T., Miki T., Goto T. (2008). Randomized prospective study comparing the laryngeal tube suction II with the proseal laryngeal mask airway in anesthetized and paralyzed patients. *Anesthesiology*.

[42] Klaver N. S., Kuizenga K., Ballast A., Fidler V. (2007). A comparison of the clinical use of the Laryngeal Tube S*™* and the ProSeal® Laryngeal Mask Airway by first-month anaesthesia residents in anaesthetised patients. *Anaesthesia*.

[43] Komatsu R., Nagata O., Kamata K., Yamagata K., Sessler D. I., Ozaki M. (2005). Comparison of the intubating laryngeal mask airway and laryngeal tube placement during manual in-line stabilisation of the neck. *Anaesthesia*.

[44] Mihai R., Knottenbelt G., Cook T. M. (2007). Evaluation of the revised laryngeal tube suction: the laryngeal tube suction II in 100 patients. *British Journal of Anaesthesia*.

[45] Noor Zairul M., Khairul Faizi A. (2006). Comparison of the VBM laryngeal tube and laryngeal mask airway for ventilation during manual in-line neck stabilisation. *Singapore Medical Journal*.

[46] Ocker H., Wenzel V., Schmucker P., Steinfath M., Dörges V. (2002). A comparison of the laryngeal tube with the laryngeal mask airway during routine surgical procedures. *Anesthesia and Analgesia*.

[47] Ozgul U., Begec Z., Karahan K. (2013). Comparison of propofol and ketamine-propofol mixture (Ketofol) on laryngeal tube-suction II conditions and hemodynamics: a randomized, prospective, double-blind trial. *Current Therapeutic Research, Clinical and Experimental*.

[48] Ratajczyk P., MaŁachowska B., Gaszyńska E., Gaszyński T. (2013). A randomised comparison between Cobra PLA and classic laryngeal mask airway and laryngeal tube during mechanical ventilation for general anaesthesia. *Anaesthesiology Intensive Therapy*.

[49] Roth H., Genzwuerker H. V., Rothhaas A., Finteis T., Schmeck J. (2005). The ProSeal laryngeal mask airway and the laryngeal tube suction for ventilation in gynaecological patients undergoing laparoscopic surgery. *European Journal of Anaesthesiology*.

[50] Russo S. G., Cremer S., Galli T. (2012). Randomized comparison of the i-gel*™*, the LMA Supreme*™*, and the Laryngeal Tube Suction-D using clinical and fibreoptic assessments in elective patients. *BMC Anesthesiology*.

[51] Schalk R., Engel S., Meininger D. (2011). Disposable laryngeal tube suction: standard insertion technique versus two modified insertion techniques for patients with a simulated difficult airway. *Resuscitation*.

[52] Thee C., Serocki G., Doerges V. (2010). Laryngeal tube S II, laryngeal tube S disposable, Fastrach laryngeal mask and Fastrach laryngeal mask disposable during elective surgery: a randomized controlled comparison between reusable and disposable supraglottic airway devices. *European Journal of Anaesthesiology*.

[53] Turan A., Kaya G., Koyuncu O., Karamanlioglu B., Pamukçu Z. (2006). Comparison of the laryngeal mask (LMA^*™*^) and laryngeal tube (LT^®^) with the new perilaryngeal airway (CobraPLA^®^) in short surgical procedures. *European Journal of Anaesthesiology*.

[54] Wrobel M., Grundmann U., Wilhelm W., Wagner S., Larsen R. (2004). Laryngeal tube versus laryngeal mask airway in anaesthetised non-paralysed patients. A comparison of handling and postoperative morbidity. *Anaesthesist*.

[55] Yildiz T. S., Solak M., Toker K. (2007). Comparison of laryngeal tube with laryngeal mask airway in anesthetized and paralysed patients. *European Journal of Anaesthesiology*.

[56] Zand F., Amini A., Sadeghi S. E., Gureishi M., Chohedri A. (2007). A comparison of the laryngeal tube-S^*™*^ and Proseal^*™*^ laryngeal mask during outpatient surgical procedures. *European Journal of Anaesthesiology*.

[57] Gruber E., Oberhammer R., Balkenhol K. (2014). Basic life support trained nurses ventilate more efficiently with laryngeal mask supreme than with facemask or laryngeal tube suction-disposable-a prospective, randomized clinical trial. *Resuscitation*.

[58] Kurola J., Pere P., Niemi-Murola L. (2006). Comparison of airway management with the intubating laryngeal mask, laryngeal tube and CobraPLA by paramedical students in anaesthetized patients. *Acta Anaesthesiologica Scandinavica*.

[59] Kurola J. O., Turunen M. J., Laakso J.-P., Gorski J. T., Paakkonen H. J., Silfvast T. O. (2005). A comparison of the laryngeal tube and bag-valve mask ventilation by emergency medical technicians: a feasibility study in anesthetized patients. *Anesthesia & Analgesia*.

[60] Schalk R., Scheller B., Habler O. P., Meier J., Meininger D., Byhahn C. (2008). Disposable laryngeal tube suction-a randomized comparison of two insertion techniques performed by novice users in anaesthetised patients. *Resuscitation*.

[61] Frascone R. J., Wewerka S. S., Burnett A. M., Griffith K. R., Salzman J. G. (2013). Supraglottic airway device use as a primary airway during rapid sequence intubation. *Air Medical Journal*.

[62] Frascone R. J., Russi C., Lick C. (2011). Comparison of prehospital insertion success rates and time to insertion between standard endotracheal intubation and a supraglottic airway. *Resuscitation*.

[63] Gahan K., Studnek J. R., Vandeventer S. (2011). King LT-D use by urban basic life support first responders as the primary airway device for out-of-hospital cardiac arrest. *Resuscitation*.

[64] Guyette F. X., Wang H., Cole J. S. (2007). King airway use by air medical providers. *Prehospital Emergency Care*.

[65] Kette F., Reffo I., Giordani G. (2005). The use of laryngeal tube by nurses in out-of-hospital emergencies: preliminary experience. *Resuscitation*.

[66] Länkimäki S., Alahuhta S., Kurola J. (2013). Feasibility of a laryngeal tube for airway management during cardiac arrest by first responders. *Resuscitation*.

[67] Müller J.-U., Semmel T., Stepan R. (2013). The use of the laryngeal tube disposable by paramedics during out-of-hospital cardiac arrest: a prospectively observational study (2008–2012). *Emergency Medicine Journal*.

[68] Russi C. S., Hartley M. J., Buresh C. T. (2008). A pilot study of the King LT supralaryngeal airway use in a rural Iowa EMS system. *International Journal of Emergency Medicine*.

[69] Schalk R., Auhuber T., Haller O. (2012). Implementation of the laryngeal tube for prehospital airway management. Training of 1,069 emergency physicians and paramedics. *Anaesthesist*.

[70] Schalk R., Byhahn C., Fausel F. (2010). Out-of-hospital airway management by paramedics and emergency physicians using laryngeal tubes. *Resuscitation*.

[71] Schalk R., Meininger D., Ruesseler M. (2011). Emergency airway management in trauma patients using laryngeal tube suction. *Prehospital Emergency Care*.

[72] Sunde G. A., Brattebø G., Ødegården T., Kjernlie D. F., Rødne E., Heltne J.-K. (2012). Laryngeal tube use in out-of-hospital cardiac arrest by paramedics in Norway. *Scandinavian Journal of Trauma, Resuscitation and Emergency Medicine*.

[73] Wiese C. H. R., Semmel T., Müller J. U., Bahr J., Ocker H., Graf B. M. (2009). The use of the laryngeal tube disposable (LT-D) by paramedics during out-of-hospital resuscitation-an observational study concerning ERC guidelines 2005. *Resuscitation*.

[74] Hubble M. W., Wilfong D. A., Brown L. H., Hertelendy A., Benner R. W. (2010). A meta-analysis of prehospital airway control techniques—part ii: alternative airway devices and cricothyrotomy success rates. *Prehospital Emergency Care*.

[75] Rai M. R., Popat M. T. (2011). Evaluation of airway equipment: man or manikin?. *Anaesthesia*.

[76] Schalk R., Seeger F. H., Mutlak H. (2014). Complications associated with the prehospital use of laryngeal tubes-a systematic analysis of risk factors and strategies for prevention. *Resuscitation*.

[77] Paal P., Timmermann A. (2014). The beauty and the beast—a tale of the laryngeal tube and related potentially life threatening operational faults. *Resuscitation*.

[78] Carlson J. N., Mayrose J., Wang H. E. (2010). How much force is required to dislodge an alternate airway?. *Prehospital Emergency Care*.

[79] Fouche P. F., Simpson P. M., Bendall J., Thomas R. E., Cone D. C., Doi S. A. R. (2014). Airways in out-of-hospital cardiac arrest: systematic review and meta-analysis. *Prehospital Emergency Care*.

[80] Barendregt J. J., Doi S. A., Lee Y. Y., Norman R. E., Vos T. (2013). Meta-analysis of prevalence. *Journal of Epidemiology and Community Health*.

[81] Deakin C. D., Murphy D., Couzins M., Mason S. (2010). Does an advanced life support course give non-anaesthetists adequate skills to manage an airway?. *Resuscitation*.

[82] Pepe P. E., Zachariah B. S., Chandra N. C. (1993). Invasive airway techniques in resuscitation. *Annals of Emergency Medicine*.

